# Effectiveness and Safety of Lenacapavir-Containing Regimens in Highly Experienced HIV-Infected Patients With Multidrug Resistance: Real-world Results From the French Compassionate Use Program ^a^

**DOI:** 10.1093/ofid/ofag353

**Published:** 2026-06-15

**Authors:** Constance Delaugerre, Sarah Mafi, Arbnor Zenuni, Gilles Peytavin, Karine Amat, Sophie Seang, Claudine Duvivier, David Chirio, Jean-Paul Viard, Laurent Hocqueloux, Emilie Estrabaud, Jade Ghosn, Roland Landman, Lambert Assoumou, Charlotte Charpentier, Karine Lacombe

**Affiliations:** Service de Virologie, AP-HP, Hôpital Saint Louis, Université Paris Cité, INSERM IRSL UMR1342, Paris, France; Service de Virologie, AP-HP, Hôpital Saint Louis, Université Paris Cité, INSERM IRSL UMR1342, Paris, France; Institut Pierre Louis d’Epidémiologie et de Santé Publique, INSERM, Université Sorbonne, Paris, France; Service de Pharmacologie, AP-HP, Hôpital Bichat-Claude Bernard, Paris, France; Institut de Médecine et d’Epidémiologie Appliquée, AP-HP, Hôpital Bichat-Claude Bernard, Paris, France; Service de Maladies Infectieuses et Tropicales, AP-HP, Hôpital Pitié-Salpêtrière, Paris, France; Service de Maladies Infectieuses et Tropicales, AP-HP, Hôpital Necker, Paris, France; Service de Maladies Infectieuses et Tropicales, Hôpital l’Archet 1, CHU de Nice, Université Côte d’Azur, Nice, France; Service de Maladies Infectieuses et Tropicales, AP-HP, Hôpital Hôtel-Dieu, Paris, France; Service de Maladies Infectieuses et Tropicales, Hôpital Universitaire d’Orléans, LI2RSO Université d’Orléans, Orléans, France; Medical Affairs France, Gilead Sciences,Paris, France; Service de Maladies Infectieuses et Tropicales, AP-HP, Hôpital Bichat-Claude Bernard, INSERM IAME, Université Paris Cité, Paris, France; Institut de Médecine et d’Epidémiologie Appliquée, AP-HP, Hôpital Bichat-Claude Bernard, Paris, France; Service de Maladies Infectieuses et Tropicales, AP-HP, Hôpital Bichat-Claude Bernard, INSERM IAME, Université Paris Cité, Paris, France; Institut Pierre Louis d’Epidémiologie et de Santé Publique, INSERM, Université Sorbonne, Paris, France; Service de Virologie, AP-HP, Hôpital Bichat-Claude Bernard, INSERM IAME, Université Paris Cité, Paris, France; Service de Maladies Infectieuses et Tropicales, AP-HP, Hôpital Saint Antoine, INSERM, Institut Pierre Louis d’Epidémiologie et de Santé Publique, UMR-S1136, Université Sorbonne, Paris, France

**Keywords:** capsid inhibitor, HIV-1, HIV-2, lenacapavir, multidrug resistance

## Abstract

**Background:**

Lenacapavir (LEN) with an optimized background regimen (OBR) was evaluated in a French cohort of participants with multidrug-resistant human immunodeficiency virus (HIV) via compassionate use.

**Methods:**

Adults (n = 42) with HIV-1 (PLWH1, n = 33) or HIV-2 (PLWH2, n = 9), receiving at least 1 dose of LEN plus OBR, were prospectively followed between January 2021 and December 2023. The primary endpoint was virological suppression (plasma HIV-1 RNA [VL] <50 copies/mL at week 26 (W26)) or maintenance, using the US Food and Drug Administration Snapshot algorithm. Secondary endpoints included long-term VL, resistance emergence, LEN plasma concentration, and drug safety.

**Results:**

At baseline, PLWH1 presented with a median VL of 4.0 log_10_ copies/mL with 14 of 33 (42%) having VL <50 copies/mL, while PLWH2 had 3.0 log_10_ copies/mL (with baseline VL <50 copies/mL for 3). By W26, virological suppression was achieved or maintained in 67.0% (95% CI, 48.2%–82.0%) of PLWH1, with a mean CD4^+^ increase of +86 cells/μL. In PLWH2, virological suppression occurred in only 22.0% (95% CI, 2.8%–60.0%), with CD4^+^ counts rising by +27 cells/μL. Emergence of LEN resistance was documented in 1 PLWH1 (Q67H) and 3 PLWH2 (all N73D). The median (IQR) LEN plasma concentration was 43 (31&ndashng/mL after 26 weeks of subcutaneous injections. Tolerance was acceptable with 31% reported injection site reactions leading to discontinuation in 2 patients, and no grade 3/4 treatment-related adverse events occurred (2 deaths unrelated to treatment).

**Conclusions:**

LEN plus OBR showed robust efficacy and safety in PLWH1 but limited antiviral activity in PLWH2 due to limited OBR. These findings support LEN as an effective option in highly treatment-experienced PLWH1, while underscoring challenges for PLWH2.

## Teaser

Lenacapavir plus an optimized regimen was effective in patients with multidrug-resistant HIV-1, but showed limited benefit in patients with HIV-2 due to fewer active drugs, while the safety profile was acceptable with mostly mild and tolerable adverse events.

Lenacapavir (LEN) is a first-in-class inhibitor of human immunodeficiency virus (HIV) capsid function with picomolar potency that exhibits antiviral activity at early and late stages of the viral life cycle [1]. LEN has been shown to bind tightly between capsid protein (CA) monomers of CA hexamers, leading to inhibited CA assembly generating displayed abnormal shapes that do not support viral replication. LEN remains broadly active in peripheral blood mononuclear cells against clinical HIV-1 variants and HIV-2 but 11- to 14-fold less potent in comparison with HIV-1 [1, 2]. LEN retains activity against HIV-1 variants resistant that are resistant to other drug classes. Furthermore, LEN has a favorable pharmacokinetic profile allowing both oral and parenteral administrations. Subcutaneous LEN can be administered twice yearly following an oral loading dose and has a minimal impact for drug interactions due to low hepatic clearance [3, 4]. Despite the availability of life-saving antiretroviral (ARV) treatments for millions of people living with HIV (PLWH), some heavily treatment-experienced (HTE) PLWH have limited or no treatment options due to multidrug resistance, tolerance, or safety concerns with other classes [5]. In addition, suboptimal adherence to daily oral regimens can negatively affect treatment outcomes, contributing to virologic failure, resistance development, and viral transmission [6, 7]. Long-acting agents from novel ARV classes can provide urgently needed treatment options for HTE PLWH and may additionally improve adherence [8].

CAPELLA (NCT04150068) was a phase 2/3, double-blind, placebo-controlled, multicenter study to evaluate the antiviral activity of LEN with optimized background regimen (OBR) in HTE PLWH with multidrug-resistant HIV-1 infection (PLWH1) [9]. The study demonstrated both the safety and antiviral efficacy of LEN in PLWH1 with limited treatment options due to preexisting resistance to at least 2 drugs per class in at least 3 of the 4 main classes of ARV drugs (nucleoside reverse transcriptase inhibitors [NRTIs], nonnucleoside reverse transcriptase inhibitors [NNRTIs], protease inhibitors [PIs], and integrase strand transfer inhibitors [INSTI]). Notably, despite approximately half of the participants exhibiting resistance to all 4 ARV classes, 81% of those who received LEN plus an OBR achieved HIV-1 RNA viral load (VL) <50 copies/mL at week (W) 26. This response was maintained through W156 [10–12]. Improved virological suppression was associated with immunological recovery, with a mean CD4^+^ increase of 97 and 122 cells/µL from baseline to W52 and W104, respectively.

Resistance to LEN was observed in 9 of 72 (13%) participants through W52 [13] and 14 of 72 (19%) through W104 [14], at amino acid residues previously identified in in vitro resistance selection experiments. Importantly, resistance emergence in CAPELLA occurred in participants with extensive baseline resistance or inadequate adherence to their OBR, resulting in functional LEN monotherapy.

In January 2021, LEN was made available in France via a French compassionate use program for viremic patients with serious or life-threatening disease or no satisfactory alternative therapy. This compassionate use program ended in December 2022 and was followed by a period of early access, and definitive approval and reimbursed for PLWH1 in August 2023. The objectives of this retrospective study were to describe virological, immunological, pharmacological, and clinical outcomes of PLWH who received LEN through the program and were followed for at least 6 months.

## METHODS

All individuals aged ≥18 years with HIV-1 (PLWH1) and/or HIV-2 (PLWH2) infection, regardless of baseline VL, who initiated LEN-based treatment between January 2021 and December 2022 were included in the IMEA-070 cohort. Data were collected from electronic medical records for participants who received LEN with an OBR and had at least 6 months of follow-up through December 2023. In December 2021, the US Food and Drug Administration (FDA) imposed a full clinical hold on subcutaneous LEN due to glass vial compatibility concerns; consequently, oral bridging (300 mg once weekly) was proposed to participants until July 2022. Genotypic drug resistance was assessed at the baseline visit and/or based on historical data on plasma RNA and cellular DNA provided by clinical investigators (Supplementary material). Changes to the OBR were permitted at the clinician's discretion.

The primary outcome was defined as the percentage of participants with suppression or maintenance of a VL <50 copies/mL after 26 weeks (W26 ± 4 weeks) of LEN-based treatment, using the FDA Snapshot algorithm. Secondary endpoints included (*i*) sustained virological control beyond W26 using the Kaplan–Meier method; (*ii*) change in CD4 cell count until the last visit of follow-up (LVFU); (*iii*) emergence of drug resistance at the time of virological failure (confirmed VL ≥50 copies/mL at W26, at discontinuation before W26, or in case of virological rebound after achieving VL <50 copies/mL); (*iv*) tolerance to LEN-based regimen; and (*v*) LEN plasma concentration according to virological outcome at the end of follow-up.

HIV-1 capsid sequencing was performed at confirmed virological failure visits. Resistance to NRTIs, NNRTIs, PIs, and INSTIs was evaluated at the same visit. Capsid inhibitor resistance-associated mutations were defined based on previous in vitro and/or in vivo studies (L56I, M66I, Q67H/K/N, K70H/N/R/S, N74D/H/K/S, A105T/S, and T107C/N) [13–15]. HIV-2 capsid sequencing and interpretation were performed as previously described [16].

LEN plasma concentrations were measured 26 weeks (±4 weeks) after an injection and before the next injection. This was performed centrally by ultra-performance liquid chromatography–tandem mass spectrometry (Acquity, Waters Corporation Milford, MA, USA), with a lower limit of quantification of 5 ng/mL. Results were interpreted using a threshold of 15 ng/mL, corresponding to 4 times the human serum protein-adjusted 95% effective concentration for wild-type HIV-1 [1]. Plasma drug concentrations of the OBR were collected for participants experiencing virological failure at W26 ± 4 weeks.

Data on adverse events (AEs) are derived exclusively from the original medical records of patients who described these events during their clinical visits.

### Statistical Analysis

Baseline clinical and demographic characteristics were described using means with standard deviation (SD) for normally distributed quantitative variables, and medians with interquartile range (IQR) for nonnormally distributed variables. Categorical variables were summarized using frequencies and percentages. The FDA Snapshot algorithm was used to assess the primary endpoint (percentage with VL <50 copies/mL at W26 ± 4 weeks with associated 95% Clopper–Pearson exact confidence intervals [CIs]). In contrast, the response at LVFU was based on available viral load data at each participant's final assessment (Kaplan–Meier method). Factors associated with virological success at W26 and up to the LVFU were explored using univariable logistic regression models for viremic PLWH1 at LEN initiation. Safety was assessed in all participants who received at least 1 dose of LEN. Informed consent permitting clinical data collection and research was obtained from all participants enrolled under the compassionate use program (IRB 2023-A02735-40).

## RESULTS

### Study Population

**Figure 1.**
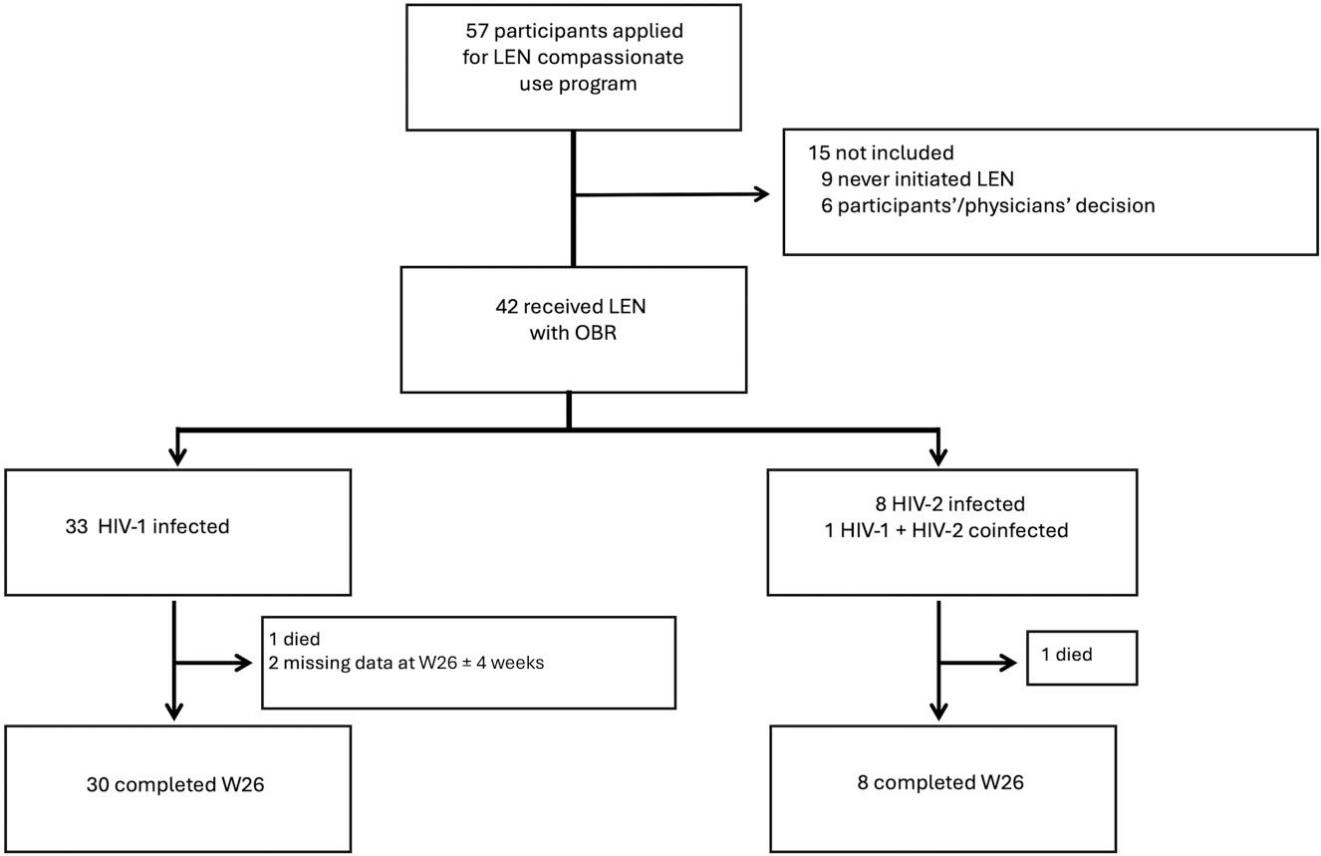
Flowchart of participants. Abbreviations: HIV-1, human immunodeficiency virus type 1; HIV-2, human immunodeficiency virus type 2; LEN, lenacapavir; OBR, optimized background regimen; W26, week 26.

The distribution of participants is shown in Figure 1. Of the 42 who received LEN with an OBR, 33 were PLWH1, and 8 were PLWH2. One participant was coinfected with HIV-1 and HIV-2 but had a controlled HIV-1 VL and was included in the PLWH2 group. The primary indications for LEN prescription were multidrug resistance (52%), virological failure (29%), drug–drug interactions (7%), difficulty with adherence (5%), and intolerance (5%) (Supplementary Figure 1). ART regimens at baseline prior to LEN initiation are described in Supplementary Figure 2 and changes made at the time of first LEN injections in Supplementary Figure 3. Initiation of LEN was associated with an ART simplification in 17 (41%), intensification in 23 (55%) and maintain in 2 (4%). Thirty individuals (71%) received 3 doses of injections of LEN 927 mg, administered subcutaneously into the abdomen, once every 26 weeks for a median duration of follow-up of 12 months (IQR, 10–15 months). Among participants who received ≥2 LEN injections (n = 37), a total of 67 LEN injections were administered. Of these, 51 (76.1%) were given within the protocol window (±2 weeks). Seven injections (10.4%) were administered early, with a median time of 23 days (IQR, 20.5–28.5 days), and 9 (13.4%) were delayed, with a median time of 141 days (IQR, 27–197 days). One PLWH2 received only oral LEN 300 mg at baseline and subsequently died.

Overall, modification of the OBR up to W26 was observed in 12 (36%) participants, principally for simplification or intensification.

### Baseline Characteristics

Baseline characteristics of patients are shown in Table 1. For the 33 PLWH1, the median age was 56 years (IQR, 41–59 years), and 33% were female. The median CD4 count was 330 cells/μL, with 11 of 27 (41%) having <200 cells/μL. Fourteen PLWH1 (42%) had VL <50 copies/mL, and the median VL was 4.0 log_10_ copies/mL for those with detectable viremia at LEN initiation. In terms of susceptibility to drugs, 13 (39%) had genotypic resistance to at least 2 drugs in all 4 major ARV classes. For entry inhibitors, resistance was detected mainly for maraviroc in 4 of 8 (50%) and for fostemsavir in 5 of 12 (42%) (Supplementary Table 1). The OBR contained a median of 3 ARVs (IQR, 3–4) (Supplementary Figure 3). The median genotypic susceptibility score (GSS) was 2, and 9 PLWH1 had only 1 active drug.

**Table 1.** Participants’ Baseline Characteristics

Characteristic		PLWH1		PLWH2^a^
	Total (N = 33)	Baseline VL <50 Copies/mL (n = 14)	Baseline VL ≥50 Copies/mL (n = 19)	(n = 9)
Age, y, median (IQR)	56 (41–59)	57 (48–60)	55 (35–58)	56 (51–60)
Sex at birth				
Male	22 (67)	12 (86)	10 (53)	4 (44)
Female	11 (33)	2 (14)	9 (47)	5 (56)
Transmission group				
Homosexual/bisexual	11 (33)	7 (50)	4 (21)	0 (0)
Heterosexual	13 (39)	5 (36)	8 (42)	7 (78)
Other	9 (27)	2 (14)	7 (37)	2 (22)
Geographic origin				
France	21 (64)	9 (65)	12 (63)	1 (11)
Sub-Saharan Africa	7 (21)	3 (21)	4 (21)	7 (78)
Other	5 (15)	2 (14)	3 (16)	1 (11)
Duration from HIV diagnosis, y, median (IQR)	28 (19–34)	31 (23–34)	27 (14–34)	20 (19–27)
Duration under ART, y, median (IQR)	25 (17–27)	26 (21–27)	24 (13–28)	20 (16–25)
Participants with VL <50 copies/mL	14 (42)	14 (100)	…	3 (33)
HIV VL, log_10_ copies/mL				
≥50 copies/mL	19 (58)	…	19 (100)	6 (67)
Median (IQR)	4.0 (2.6–5.1)	…	4.0 (2.6–5.1)	3.0 (1.6–3.2)
CD4 count, cells/μL	n = 27	n = 11	n = 16	n = 9
Median (IQR)	330 (106–500)	464 (129–714)	278 (72–444)	140 (95–247)
<200 cells/μL	11 (41)	4 (36)	7 (44)	6 (67)
Clinical stage C	18 (55)	8 (57)	10 (53)	3 (33)
Viral subtype/group				
HIV-1 subtype B	23 (70)	11 (79)	12 (63)	…
HIV-1 subtype non-B	10 (30)	3 (21)	7 (27)	…
HIV-2 group A	…	…	…	5 (56)
HIV-2 group B	…	…	…	4 (44)
Resistance to ≥2 drugs in major class				
NRTI	27 (82)	10 (71)	17 (89)	9 (100)
NNRTI	30 (91)	12 (86)	18 (95)	9 (100)
PI	16 (49)	8 (57)	8 (42)	8 (89)
INSTI	20 (61)	9 (64)	11 (58)	7 (78)
All 4 major classes	13 (39)	7 (50)	6 (32)	6 (67)
Composition of OBR^b^				
NRTI	14 (45)	7 (50)	7 (37)	9 (100)
NNRTI	9 (27)	5 (36)	4 (21)	…
PI	12 (36)	4 (29)	8 (42)	9 (100)
INSTI	21 (64)	11 (79)	10 (53)	8 (89)
Enfuvirtide	4 (12)	1 (7)	3 (16)	…
Fostemsavir	12 (36)	4 (29)	8 (42)	…
Ibalizumab	7 (21)	2 (14)	5 (26)	4 (44)
Maraviroc	8 (24)	3 (21)	5 (42)	2 (22)
Foscarnet^c^	2 (6)	1 (7)	1 (5)	1 (11)
Overall susceptibility score of OBR, median (IQR)^d^	2 (1–2.5)	1.75 (1–2)	2 (1–3)	1 (1–1.5)
No. of fully active agents in the OBR				
0–0.5	2 (6)	0 (0)	2 (11)	0 (0)
≥1–1.5	11 (33)	7 (50)	4 (21)	7 (78)
≥2	20 (61)	7 (50)	13 (68)	2 (22)

Data are presented as No. (%) unless otherwise indicated.

Abbreviations: ART, antiretroviral therapy; HIV, human immunodeficiency virus; IQR, interquartile range; INSTI, integrase strand transfer inhibitor; NNRTI, nonnucleoside reverse transcriptase inhibitor; NRTI, nucleoside reverse transcriptase inhibitor; OBR, optimized background regimen; PI, protease inhibitor; PLWH1, people living with human immunodeficiency virus type 1; PLWH2, people living with human immunodeficiency virus type 2; VL, viral load.

^a^One participant was coinfected with HIV-1 and HIV-2 but with a controlled HIV-1 VL, so he was described with the PLWH2 participants.

^b^Of the 33 PLWH1 participants, 7 (21%) received dolutegravir twice a day and 9 (27%) received boosted darunavir twice a day. Of the 9 PLWH2 participants, 2 (22%) received dolutegravir twice a day and 5 (56%) received boosted darunavir twice a day.

^c^Intravenous foscarnet added to a failing regimen in late-stage HIV-1 with triple-class resistance showed a modest viro-immunological effect (Canestri et al, Antiviral Therapy 2006, https://doi.org/10.1177/135965350601100501).

^d^Genotypic susceptibility scores (1 for full, 0.5 for possible resistance, or 0 for full resistance) were determined using the Agence Nationale de Recherche sur le SIDA French algorithm V35 to interpret cumulated historical resistance reports (https://hivfrenchresistance.org/). The overall susceptibility score of the OBR was the sum of the individual scores.

For the 9 PLWH2, the median age was 56 years (IQR, 51–60 years), and 56% were female. At LEN initiation, the median CD4 count was 140 cells/μL, with 6 (67%) having <200 cells/μL. Three PLWH2 (34%) had VL <50 copies/mL at initiation of LEN-based therapy, and for those with virological failure, the median VL was 3.0 log_10_ copies/mL. Cumulative genotypic resistance analysis revealed that 100%, 89%, and 78% of PLWH2 harbored viruses resistant to at least 2 drugs in the NRTI, PI, and INSTI drug classes, respectively. Six PLWH2 (67%) had resistance to at least 2 drugs in all 4 major classes. The OBR included a median of 5 ARVs (IQR, 4–5) (Supplementary Figure 2). The median GSS was 1.0, and 4 PLWH2 had only 1 fully active drug.

### Clinical Outcomes

**Figure 2.**
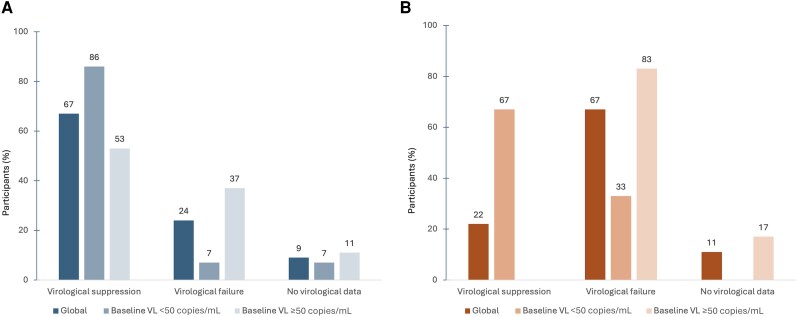
Virologic outcomes at W26 in PLWH1 and PLWH2 receiving a lenacapavir-based regimen. Percentage of PLWH1 (**A** in blue) and of PLWH2 (**B** in orange) who achieved or maintained plasma HIV-1 RNA <50 copies/mL at W26 according to the US Food and Drug Administration Snapshot algorithm. PLWH1 and PLWH2 with baseline VL <50 copies/mL are shown in light blue and orange and those with baseline VL ≥50 copies/mL in very light blue and orange. Abbreviations: HIV-1, human immunodeficiency virus type 1; PLWH1, people living with human immunodeficiency virus type 1; PLWH2, people living with human immunodeficiency virus type 2; VL, viral load; W26, week 26.

**Figure 3.**
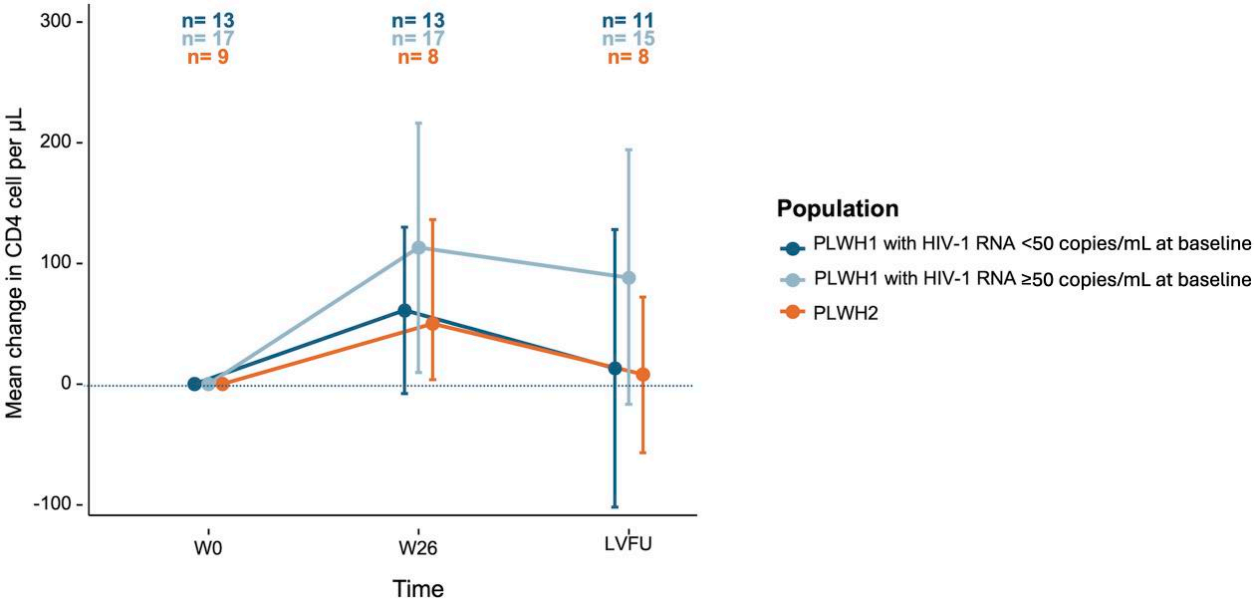
Mean change in CD4^+^ cell count from baseline. PLWH1 are shown according to baseline plasma HIV-1 RNA: <50 copies/mL (dark blue) and ≥50 copies/mL (light blue). PLWH2 are shown in orange. Bars indicate 95% confidence interval. Abbreviations: HIV-1, human immunodeficiency virus type 1; LVFU, last visit of follow-up; PLWH1, people living with human immunodeficiency virus type 1; PLWH2, people living with human immunodeficiency virus type 2; W0, week 0; W26, week 26.

At W26 ± 4 weeks, a VL <50 copies/mL was observed in 67% (95% CI, 48%–82%) of the PLWH1 (Figure 2). The mean change in VL was −1.19 (SD, 1.49) log_10_ copies/mL. Virological failure occurred in 8 of 33 (24%) participants (median VL 118 (IQR 75-167) copies/ml), and data were missing for 3 (1 death, 2 missing records in the assessment window). Among PLWH1 with baseline VL <50 copies/mL, 86% (12/14) remained controlled at W26, and 7% (1/14) experienced loss of suppression (VL >50 copies/mL) by W26. Among those who were viremic at baseline (VL ≥50 copies/mL), 53% (10/19) achieved viral suppression. W26 virological success rates were higher in PLWH1 aged >50 years and those with virus resistant to at least 2 PIs at LEN initiation (Table 2). The sustained virological control after W26 is described in Supplementary Figures 4 and 5. For baseline viremic and suppressed PLWH1, 74% and 92% had VL <50 copies/mL at month 12, respectively. Among the 33 PLWH1 with CD4 results, the mean increase from baseline to W26 was +86 cells/μL (IQR, 10–197 cells/μL), and the proportion with CD4 >200 cells/μL rose from 60% to 68%. At LVFU, the mean increase was 51 cells/μL (95% CI, −1 to 168 cells/μL), with 81% >200 cells/μL (Figure 3). Capsid gene sequencing was performed in 6 of 8 individuals with virological failure, revealing 1 participant with treatment-emergent LEN resistance (Q67H at W16, Supplementary Table 2). Among 5 patients with genotypes available, no new mutations were found in other genes.

**Table 2.** Associated Factors With the Week 26 Virological Response Among the 19 People Living With HIV-1 Who Were Viremic at Baseline (Univariate Analysis)

Characteristic	No.	Event	OR (95% CI)	*P* Value
Age				
<50 y	7	3	1.00	
≥50 y	12	11	14.7 (1.50–356)	.04
Sex				
Female	9	5	1.00	
Male	10	5	0.80 (.12–4.94)	.81
Transmission group				
Homosexual/bisexual	4	4	1.00	
Heterosexual	8	4	0.00	.99
Other	7	2	0.00	.99
Geographic origin				
Sub-Saharan Africa	4	2	1.00	
Other	3	1	0.50 (.01–10.9)	.66
France	12	7	1.40 (.13–15.3)	.77
Viral subtype				
B	12	7	1.00	
Non-B	7	4	0.27 (.03–2.19)	.22
Baseline viral load				
<4 log_10_ copies/mL	10	6	1.00	
≥4 log_10_ copies/mL	9	4	0.53 (.08–3.27)	.49
Baseline CD4 count (n = 16)				
<200 cells/µL	7	2	1.00	
≥200 cells/µL	9	7	8.75 (1.05–113)	.06
Resistance to least 2 protease inhibitors				
No	10	3	1.00	
Yes	9	7	8.17 (1.17–83.4)	.05
Resistance to least 2 integrase inhibitors				
No	8	3	1.00	
Yes	11	7	2.92 (.46–21.7)	.07
Overall susceptibility score				
0–1.5	6	5	1.00	
≥2	13	9	0.45 (.02–4.22)	.52
No. of fully active antiretroviral agents				
<2	6	5	1.00	
≥2	13	9	0.45 (.02–4.22)	.52
Use of ibalizumab and/or fostemsavir				
No	8	5	1.00	
Yes	11	9	2.70 (.34–26.5)	.35

Abbreviations: CI, confidence interval; OR, odds ratio.

For the 9 PLWH2, VL <50 copies/mL was achieved or maintained in 22% (95% CI, 2.8%–60.0%) at W26 (Figure 2). Virological failure was seen in 6 of 9 (67%). None of the baseline viremic PLWH2 achieved control at W26, while 2 of 3 controlled at baseline maintained it. At LVFU, 2 of 9 (22% [95% CI, 3%–60%]) had a VL <50 copies/mL. Individual evolution of viral load is presented in Supplementary Figure 6. Among the 9 PLWH2 with CD4 results, the median increase from baseline to W26 was +27 cells/μL (IQR, −12 to +138 cells/μL), and the proportion with CD4 >200 cells/μL increased from 38% to 63%. At LVFU, the median increase from baseline was +12 cells/μL (IQR, −35 to +35 cells/μL). Among the 6 PLWH2 with virological failure, capsid sequencing indicated that LEN resistance mutations emerged in 3 of 6 cases at early time points (W8, W16, and W20), each with the N73D mutation, as previously described [16].

**Figure 4.**
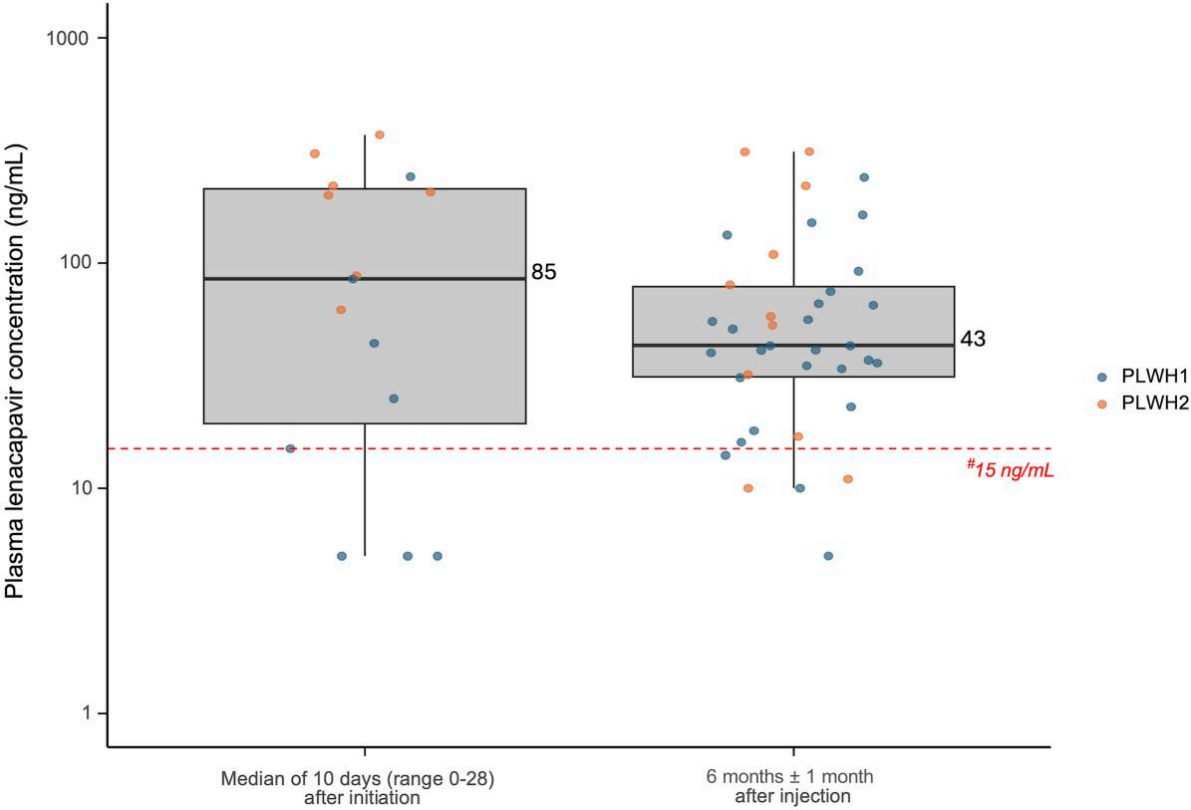
Plasma concentration (ng/mL) of LEN during follow-up (median and interquartile range). The first boxplot represents the concentration after a median of 10 days after initiation of oral LEN. The second boxplot represent the concentration after 6 months (± 4 weeks) of LEN injection. PLWH1 are shown in blue, PLWH2 in orange. ^#^15 ng/mL = 4-fold protein-adjusted 95% effective concentration on wild-type HIV-1 viruses. Abbreviations: HIV-1, human immunodeficiency virus type 1; LEN, lenacapavir; PLWH1, people living with human immunodeficiency virus type 1; PLWH2, people living with human immunodeficiency virus type 2.

All 42 participants received oral LEN at treatment initiation. Among the 33 PLWH1, 4 (12%) received a single injection, 5 (15%) a second, and 24 (73%) a third. Of the 9 PLWH2, 1 completed oral dosing but did not receive injection, 2 (22%) received a second injection, and 6 (67%) a third. Four participants received oral bridging. Thirty-eight plasma samples were collected from 27 patients (20 PLWH1, 7 PLWH2) (Figure 4 and Supplementary Figure 7). The median (IQR) plasma LEN concentration was 85 ng/mL (15–220 ng/mL) approximately 10 days after the last oral LEN dose and 43 ng/mL (31–80 ng/mL) 26 weeks (±4 weeks) after the last LEN subcutaneous injection and before the next one, with no difference by virological outcome at W26 (Supplementary Figure 7). Among PLWH who failed, plasma concentrations of OBR were obtained for 12 and considered as adequate for 7 (Supplementary Table 2). Among the 10 with adequate LEN concentration, 3 had inadequate drug level of OBR drugs, suggesting a difference of adherence between oral and injectable drugs.

Table 3 lists AEs reported during follow-up. Two patients died: 1 PLWH1 at W15 due to septic shock and 1 PLWH2 at W4 due to flare-up after hepatitis B virus reactivation. Neither death was deemed related to LEN. Thirteen participants (31%) experienced at least 1 study drug–related injection site reaction, including subcutaneous nodule (12/42 [28%]), pain (10/42 [24%]), swelling (3/42 [7%]), redness (1/42 [2%]), and abdominal cellulitis (1/42 [2%]), leading to discontinuation in 2 patients. The most frequent non–injection site AEs were fatigue (4/42 [10%]), diarrhea (3/42 [7%]), and nausea/vomiting (3/42 [7%]), mostly mild to moderate. Eleven of 42 (26%) had grade 3 or higher AEs, none considered LEN-related.

**Table 3.** Adverse Events in People Living With HIV-1 or HIV-2 During Follow-up

Adverse Event	LEN (n = 42)
Injection site reaction^a^	
Nodule	12 (28)
Pain	10 (24)
Swelling	3 (7)
Redness	1 (2)
Abdominal cellulitis	1 (2)
Any AE, excluding injection site reactions^b^	
Fatigue	4 (10)
Diarrhea	3 (7)
Nausea/vomiting	3 (7)
AE grade 3 or higher^b^	11 (26)
LEN related	0 (0)

Data represent No. (%) of participants. Multiple AEs were counted only once per patient for the highest-severity grade for each preferred term.

Abbreviations: AE, adverse event; LEN, lenacapavir.

^a^Most injection site reactions, including pain, were grade 1 or 2.

^b^AE occurring in at least 5% of participants.

## DISCUSSION

After the initial results of the CAPELLA trial that reported improved rates of virologic suppression of LEN combined with OBR in patients failing ARV regimen with multidrug-resistant HIV-1 infection [9–12], LEN became available in France through a compassionate use program. Our study focuses on the characterization of patients included in this program and we described, for the first time outside of a trial, their outcomes after at least 6 months on a LEN-based regimen. We included 42 PLWH, comprising 33 with HIV-1 and 9 with HIV-2 (mono- or coinfected) who harbored multidrug-resistant viruses. Among the 33 PLWH1, 19 were not controlled at baseline and may be comparable with PLWH1 included in the CAPELLA study. Moreover our study is the first to report outcomes in controlled PLWH1 switching to LEN-based therapy, and also provides comprehensive data for PLWH2, presented elsewhere [16].

We found baseline characteristics of PLWH1 in our cohort similar to those in the CAPELLA trial (even in those with controlled patients at baseline), with a median age of 56 versus 55 years, a proportion of females of 33% versus 25%, and a baseline viral load of 4 log_10_ copies/mL for viremic patients. CD4 cell counts were lower in CAPELLA patients (60% vs 41% with <200 cells/μL). Both studies found extended multidrug resistance, with 39% of patients from the French program and 46% of CAPELLA patients showing resistance to at least 2 drugs from all 4 major ARV classes. The OBR regimens differed in the French study and in CAPELLA in terms of NRTI use (45% vs 85%), NNRTI use (27% vs 35%), and PI use (36% vs 63%), with INSTI and entry inhibitor usage being similar. French patients were more likely to receive an OBR containing at least 2 fully active agents compared to CAPELLA (61% vs 47%).

Regarding virological efficacy, a VL <50 copies/mL at W26 was reported globally in 67% PLWH1 using the FDA Snapshot algorithm. For participants viremic at baseline (VL ≥50 copies/mL), 53% achieved a VL <50 copies/mL at W26 and 74% during the follow-up period of 12 months. LEN-emergent resistance was observed in only 1 patient who discontinued before W26. Our findings contrast slightly with the overall CAPELLA results, where 81% of patients achieved viral suppression at W26 [9], but with similar suppression at W52 (78% VL <50 copies/mL [10]). As virological failure in CAPELLA was defined as a confirmed VL at least 50 copies/mL and a decrease of <1 log_10_ copies/mL at W4 after LEN initiation, the level of VL was higher and associated with a greater incidence of LEN-related resistance [13, 14]. The slightly lower response at W26 in the French cohort is likely explained by a stricter definition of virological failure. In fact, most of French PLWH1 failing treatment had a VL <200 copies/mL with an optimized OBR (high GSS at baseline or intensified during follow-up) with no emergence of resistance mutation. Among participants already suppressed at LEN initiation (defined as VL <50 copies/mL at baseline), the objective is to maintain the virological suppression after the switch to LEN-based therapy. We have demonstrated a high level of suppression at W26 (86%) and beyond (92%). It should be noted that LEN-based therapy was chosen in order to simplify the ARV combination (around 40%), either by using fewer oral medications or by removing certain ARVs. Globally, the level of LEN and OBT is adequate, suggesting a small difference of adherence between oral and injectable drugs as previously described in the CAPELLA trial.

As demonstrated in the CAPELLA study, restoration of CD4 cell counts was observed in these PLWH1, especially among those who were viremic at baseline.

For virological outcomes in PLWH2 receiving LEN, our previous report indicated that despite initial efficacy, sustained virological suppression was not achieved after 1 year; 5 participants developed capsid drug-resistance mutations, including N73D in 4 cases [16]. In the present analysis, we report outcomes for all 9 PLWH2 treated in the French compassionate use program, 7 of whom were described in greater detail previously [16]. Virological failure was common among PLWH2 and associated with low GSS, as 4 had only 1 fully active drug in addition to LEN. HIV-2 drug resistance mechanisms are known to confer resistance more frequently and rapidly, including for the PI drug class [17]. It is plausible that similar mechanisms may occur with capsid inhibitors, particularly when used as salvage regimen in highly treatment experienced PLWH2. Moreover, plasma LEN concentrations were similar in PLWH1 and PLWH2, although the inhibitory concentration of 95% for HIV-2 is approximately 10-fold higher [1, 2]. But virological failure occurred rapidly and probably before the pharmacokinetic steady state of LEN was reached. This reduced in vitro potency against HIV-2 may explain the lower virological response in fully adherent patients, as confirmed by therapeutic drug monitoring of other ARVs in OBR.

Most AEs were mild or moderate, including injection site reactions. LEN discontinuation occurred in 2 participants, primarily due to fatigue.

The main limitation of this study is its retrospective design, with no dedicated visits to monitor AEs or safety signals, increasing the risk of misinterpretation. Due to the small sample size of viremic patients, we did not find expected associated factors to success as the number of fully active drug in OBR. However, the highly treatment experienced population with multidrug resistance is small, and it is challenging to assess new drugs or therapeutic classes in this setting. This also makes it difficult to analyze the effect of LEN alone as physicians changed OBT during the follow-up to avoid a virologic failure in these patients with multidrug resistance and no therapeutic options. In contrast, there are more controlled individuals with resistant viruses and a long history of treatment failure who require potent switching therapies. The sample size for PLWH2 is also small, but it provides information to guide clinical decisions.

In conclusion, LEN combined with an OBR was well tolerated and showed improved rates of virologic suppression in PLWH1 harboring multidrug-resistant virus with limited therapeutic options. Efficacy was higher among controlled participants who switched to LEN. In contrast, virological control remains difficult in PLWH2, as the number of susceptible ARVs to combine with LEN is very limited.

## Supplementary Material

ofag353_Supplementary_Data

## Supplementary Data

Supplementary materials are available at *Open Forum Infectious Diseases* online. Consisting of data provided by the authors to benefit the reader, the posted materials are not copyedited and are the sole responsibility of the authors, so questions or comments should be addressed to the corresponding author.
