# Supplementary material for: Effectiveness and Safety of Lenacapavir-Containing Regimens in Highly Experienced HIV-Infected Patients With Multidrug Resistance: Real-world Results From the French Compassionate Use Program
Source: Open Forum Infect Dis. 2026 Jun 15;13(6):ofag353. doi: 10.1093/ofid/ofag353 (PMC13280643; doi:10.1093/ofid/ofag353)
Supplement: ofag353_Supplementary_Data [file ofag353_supplementary_data.docx]

**Supplemental files**

**Section Methods**

Cumulative historical resistance genotypes were assessed for NRTI, NNRTI, PI, and INSTI. Entry inhibitor susceptibility was tested only for participants who received these drugs in their OBR. In cases of envelope sequencing failure, prior virological failure to that drug was considered as due to resistance (Table S1). Genotypic susceptibility scores (1 for full susceptibility, 0.5 for possible resistance, or 0 for full resistance) were determined using the ANRS French algorithm V35 (www.hivfrenchresistance.org). The overall OBR susceptibility score was calculated as the sum of the individual drug scores.

**Table S1: Baseline genotypic susceptibility score of the optimized background regimen**

The level of drug-resistance according to the cumulative historical resistance genotypes for nucleoside reverse transcriptase inhibitors (NRTI), non-nucleoside reverse transcriptase inhibitors (NNRTI), protease inhibitors (PI) and integrase strand-transfer inhibitors (INSTI) was determined using the French resistance algorithm ANRS V35 (<https://hivfrenchresistance.org/>).

Entry inhibitors susceptibility was tested only for participants who have received these drugs.

Fostemsavir resistance was done on the sequence of the gp120 gene and interpreted according to the French resistance algorithm ANRS V35.

In case of sequencing failure, the drug was considered resistant if a prior virological failure had occurred under that drug (suppl Table S1). Foscarnet was considered to be active.

The genotypic susceptibility scores (GSS) at baseline were calculated for each drug of the optimized background regimen (OBR) (1 for full, 0.5 for intermediate, or 0 for no susceptibility) except the lenacapavir. The overall GSS was the sum of the individual scores.

Green: sensitive drug (=1)

Orange: possible resistance (=0.5)

Red: probable resistance (=0)

**S1a: for PLWH1**

**
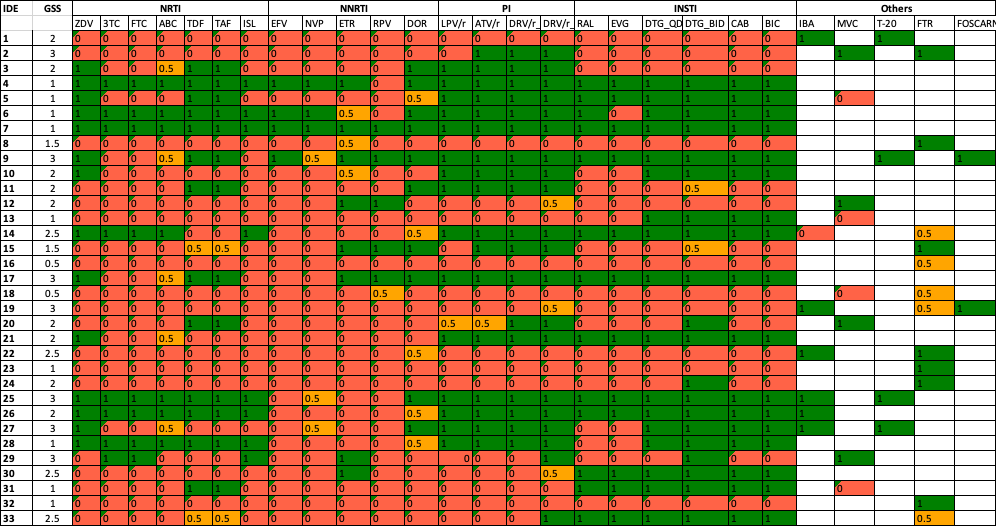
**

**S1b: for PLWH2**

**Section results**

**Figure S1 : Indications for LEN use**

**
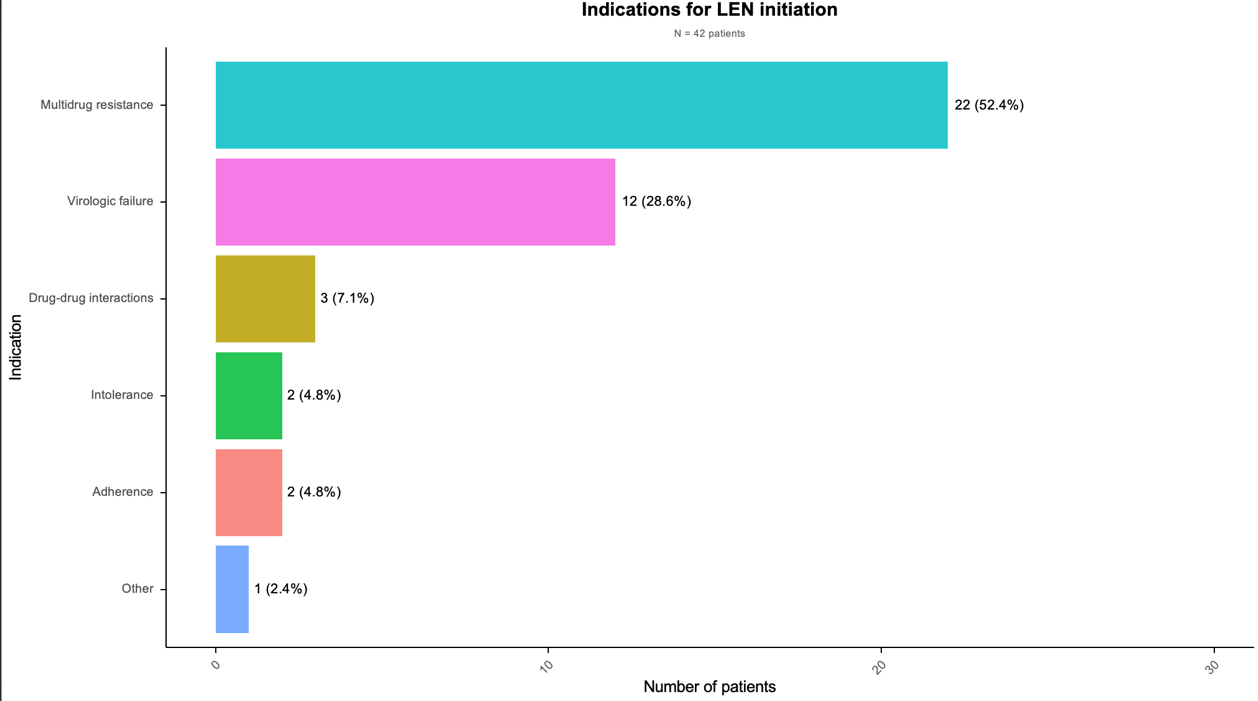
**

**Figure S2 : ART regimens at baseline prior to LEN initiation (number of patients)**

**Figure S3 : Changes made at the time of first LEN injections (% of ARV used)**

**Figure S4: Cumulative probability of achieving VL < 50 copies/mL among the 19 PLWH1 with baseline viremic VL**

**
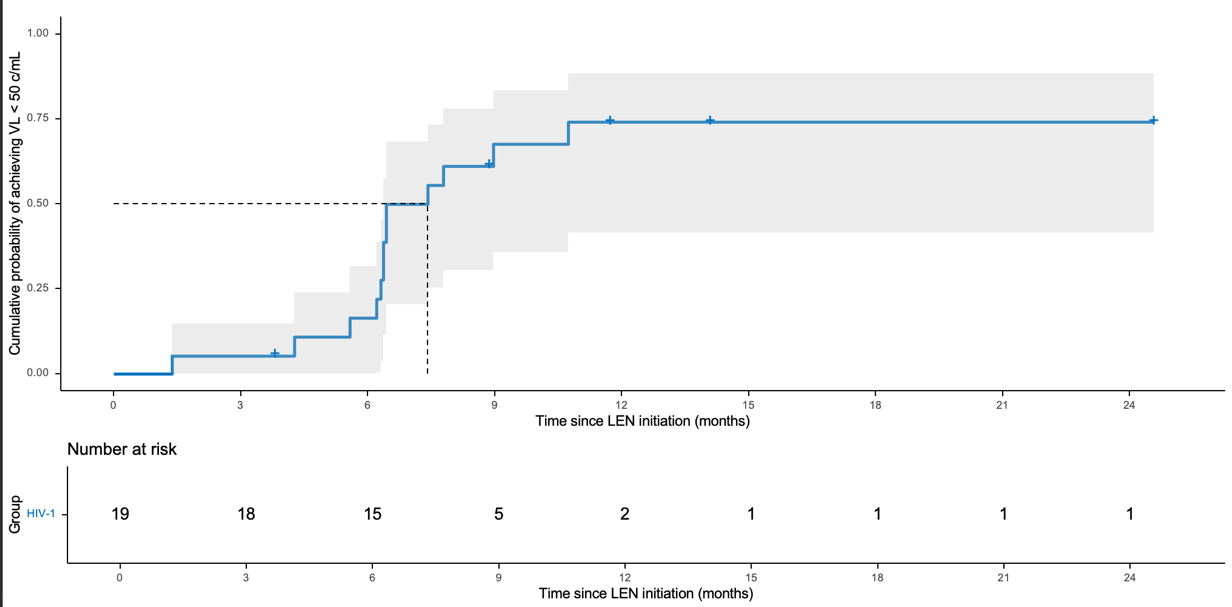
**

**Figure S5: Cumulative probability of maintaining VL < 50 copies/mL among the 14 PLWH1 with baseline controlled VL**

**
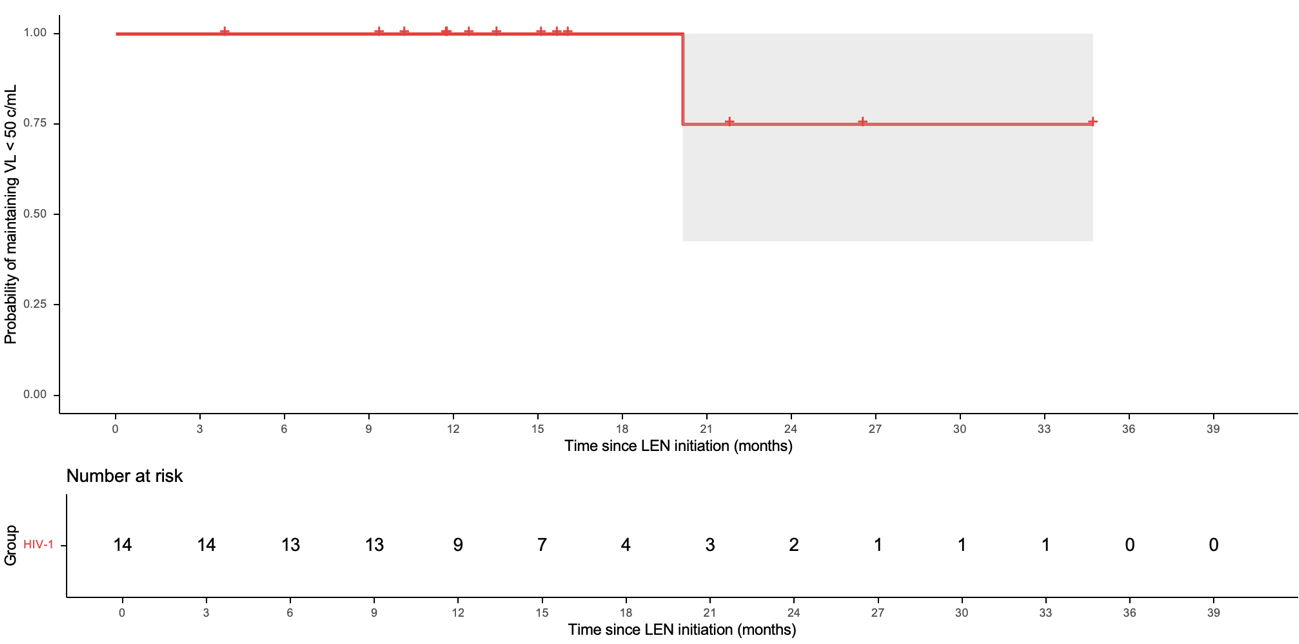
**

**Figure S6: individual VL evolution for PLWH2**

*
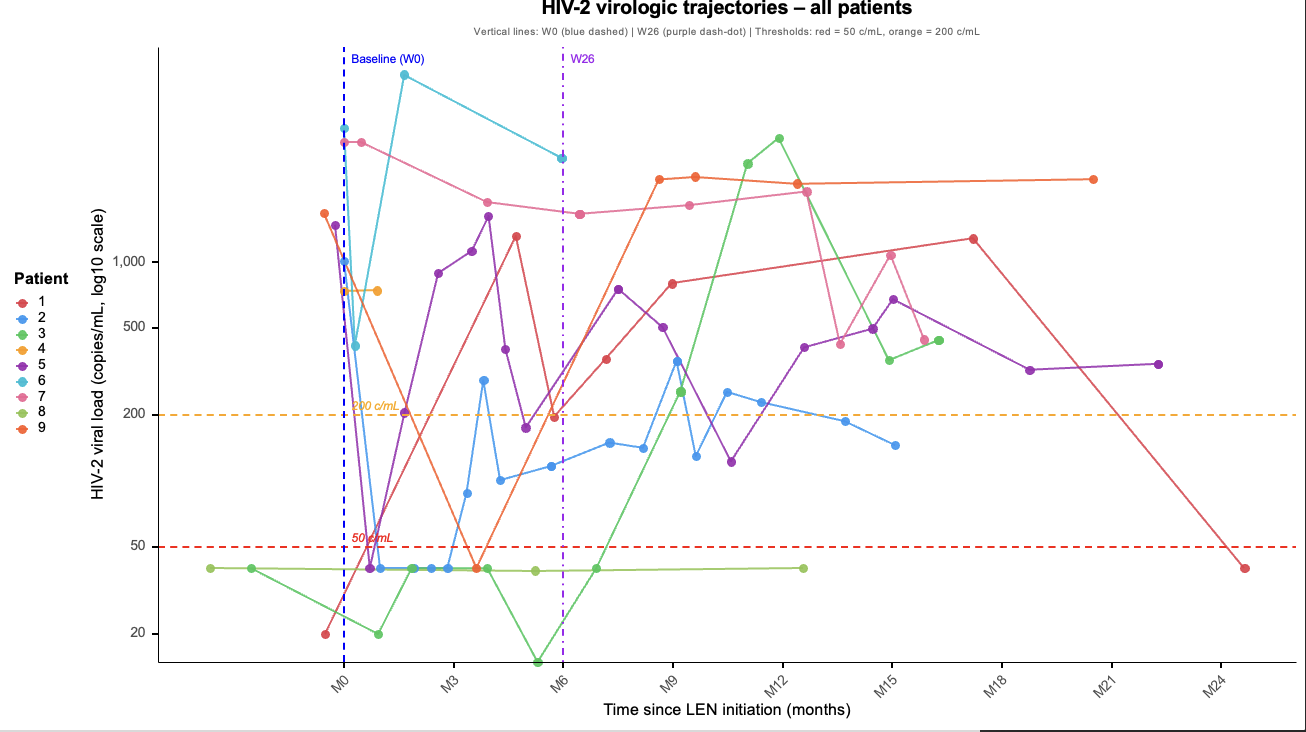
*

**Table S2 : Lenacapavir resistance and plasma ARV concentrations in participants with virological failure at W26**

**Figure S7 : Plasma concentration of lenacapavir according to virological failure at W26**
